# Supplementary material for: Cathepsin-dependent amyloid formation drives mechanical rupture of lysosomal membranes
Source: bioRxiv. 2026 Jan 19:2026.01.17.700056. Preprint. [Version 1] doi: 10.64898/2026.01.17.700056 (PMC12871838; doi:10.64898/2026.01.17.700056)
Supplement: 1 [file NIHPP2026.01.17.700056V1-supplement-1.pdf]

bioRxiv preprint doi: https://doi.org/10.64898/2026.01.17.700056; this version posted January 19, 2026. The copyright holder for this preprint (which was not certified by peer review) is the author/funder, who has granted bioRxiv a license to display the preprint in perpetuity. It is made available under aCC-BY 4.0 International license.

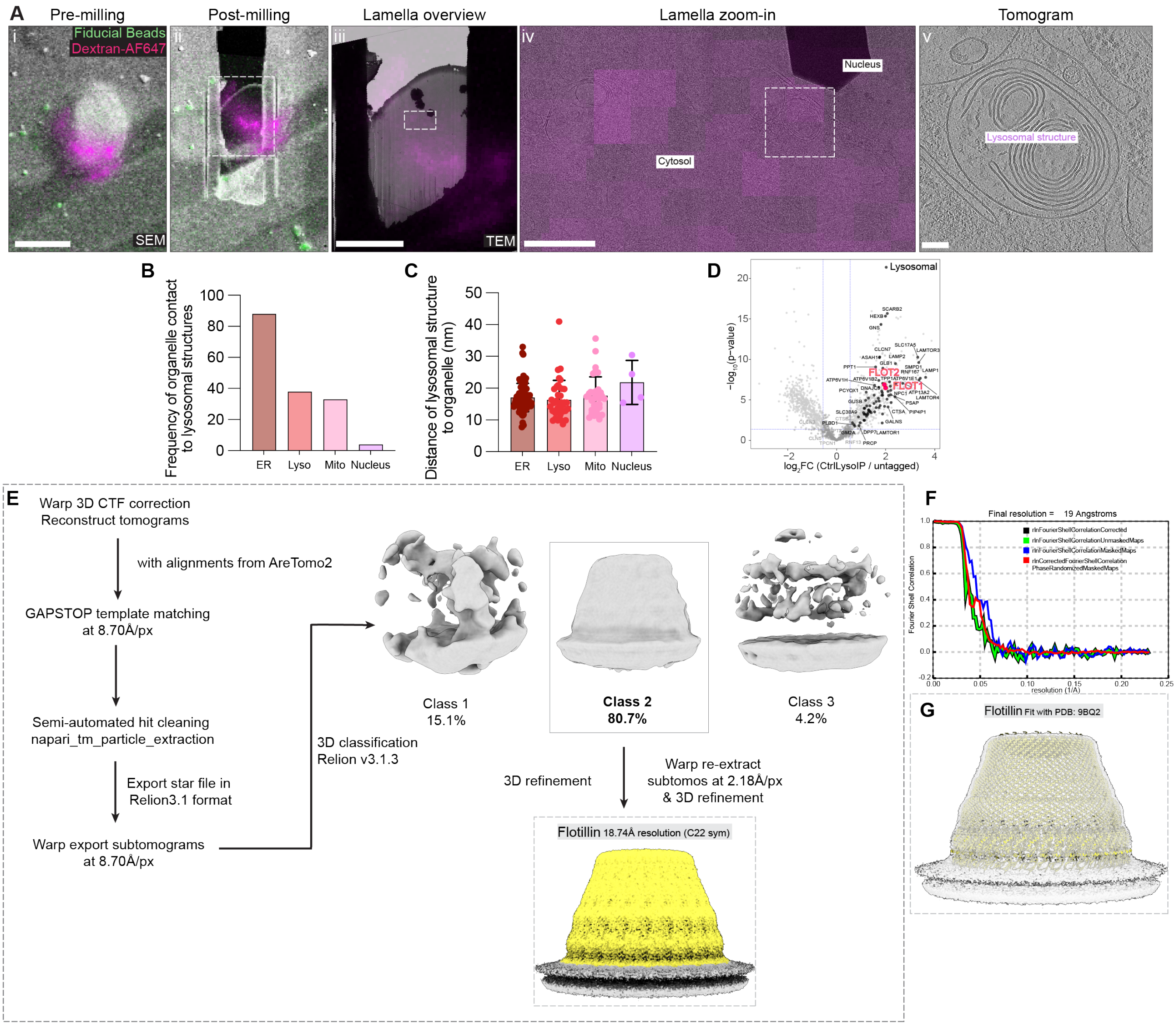

Fig. S1. In situ investigation of lysosomes in untreated HeLa cells.
(**A**) Correlative workflow to target endo-lysosomal structures in HeLa cells vitrified on cryo-EM grids. (i) maximum intensity projection overlay of Dextran-AF647 and fiducial beads fluorescence with SEM image. Scale: 20 μm (ii) Pre-milling fluorescence overlaid with final post-milling lamella SEM image. (iii) Lamella overview montage image from TEM overlaid with pre-milling fluorescence image. Distinction of cytosol/nucleus are indicated Scale: 10 μm. (iv) Zoom-in into the lamella map with tilt series acquisition area outlined. Scale: 1 μm. (**B**) Manual counting of organelle contacts with untreated lysosomes within 50 nm (**C**) Manual measurements of organelle–lysosome distances (n=65 tomograms). Median and 95% confidence interval (CI) is plotted. (**D**) Volcano plot of HeLa Lyso-IP proteomics (log_2FC vs p-value) with lysosomal proteins marked in black and Flotillin complex (FLOT1/2) in red. (**E**) Workflow for template matching and subtomogram averaging of Flotillin complex in situ. Software versions used are indicated. (**F**) Fourier shell correlation plot from Relion output demonstrating a final resolution of 19 Å for the Flotillin cryo-ET map. (**G**) Fit of cryo-ET map (in transparent) with published atomic model of Flotillin complex PDB: 9BQ2 (*84*).

bioRxiv preprint doi: https://doi.org/10.64898/2026.01.17.700056; this version posted January 19, 2026. The copyright holder for this preprint (which was not certified by peer review) is the author/funder, who has granted bioRxiv a license to display the preprint in perpetuity. It is made available under aCC-BY 4.0 International license.

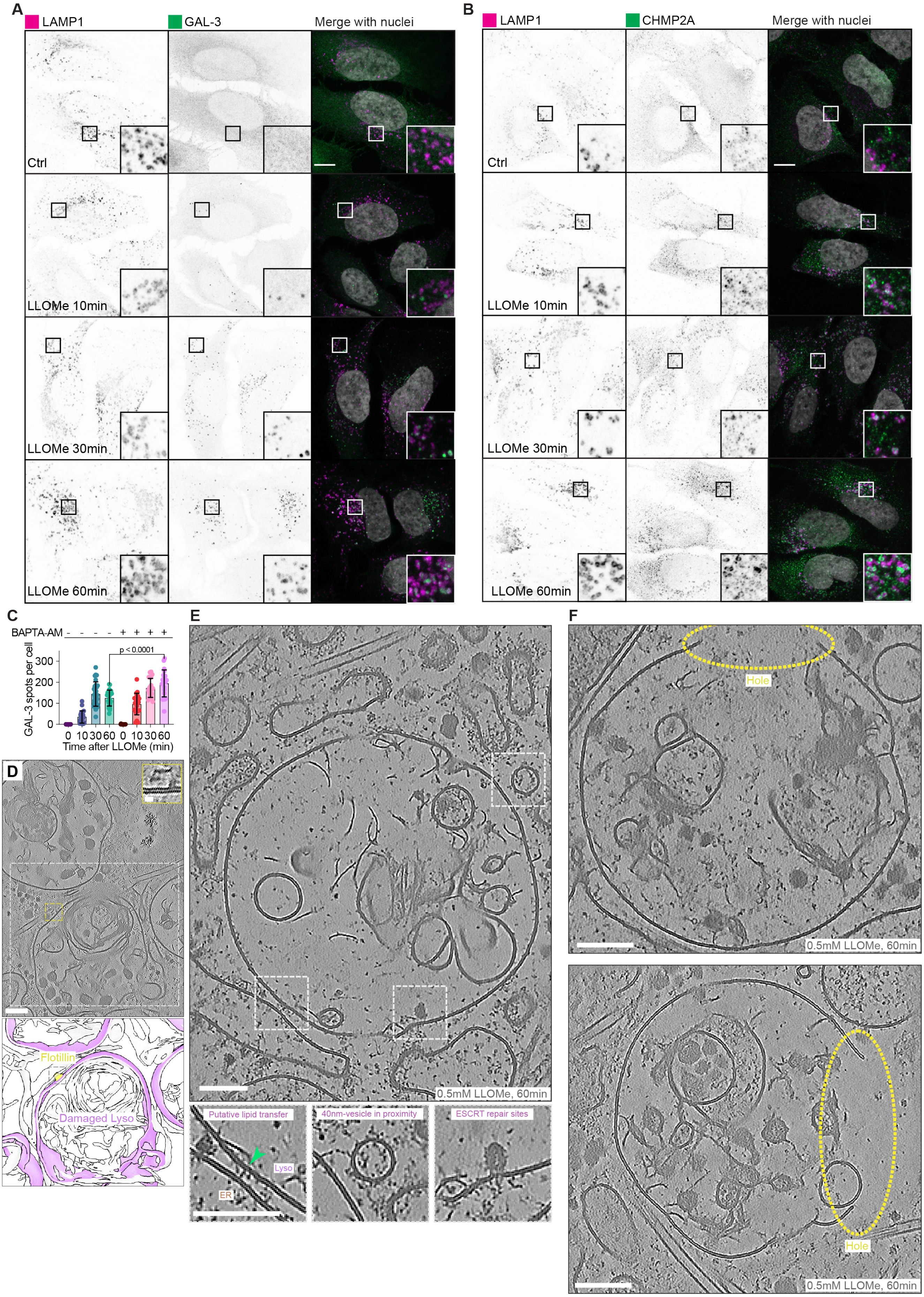

bioRxiv preprint doi: https://doi.org/10.64898/2026.01.17.700056; this version posted January 19, 2026. The copyright holder for this preprint (which was not certified by peer review) is the author/funder, who has granted bioRxiv a license to display the preprint in perpetuity. It is made available under aCC-BY 4.0 International license.

Fig. S2. Cellular responses after LLOMe treatment.
(**A**) Confocal images of immunofluorescence against LAMP1 (lysosome) and GAL-3 (lysosomal damage marker). Split channels and a merged image with Hoechst (nucleus) are shown. Untreated control and timepoints (10, 30, 60 min) after LLOMe treatment (0.5 mM) are indicated. (**B**) Same timepoints and treatments as in A but stained for CHMP2A. Scale for IF panels: 10 μm. (**C**) Quantification of GAL-3 and CHMP2A spots per cell across time course. BAPTA-AM pre-treated conditions are indicated (mean ± s.d.). Statistical test: Unpaired t-test with p-values indicated. (**D**) Tomographic slice of LLOMe-treated lysosome with segmentation and Flotillin coordinates placed back in. Zoom-in showing Flotillin in tomographic slice. Scale: 10 nm for zoom-in, 100 nm for tomographic slice. (**E**) Tomographic slice shown in Fig. 1F with zoom-ins of putative membrane repair mechanisms (lipid transfer, 40 nm ATG9A vesicles, ESCRT budding). Scale: 100 nm. (**F**) Tomographic slice of lysosomes in LLOMe-treated cells with apparent holes (circled in yellow). Scale: 100 nm.

bioRxiv preprint doi: https://doi.org/10.64898/2026.01.17.700056; this version posted January 19, 2026. The copyright holder for this preprint (which was not certified by peer review) is the author/funder, who has granted bioRxiv a license to display the preprint in perpetuity. It is made available under aCC-BY 4.0 International license.

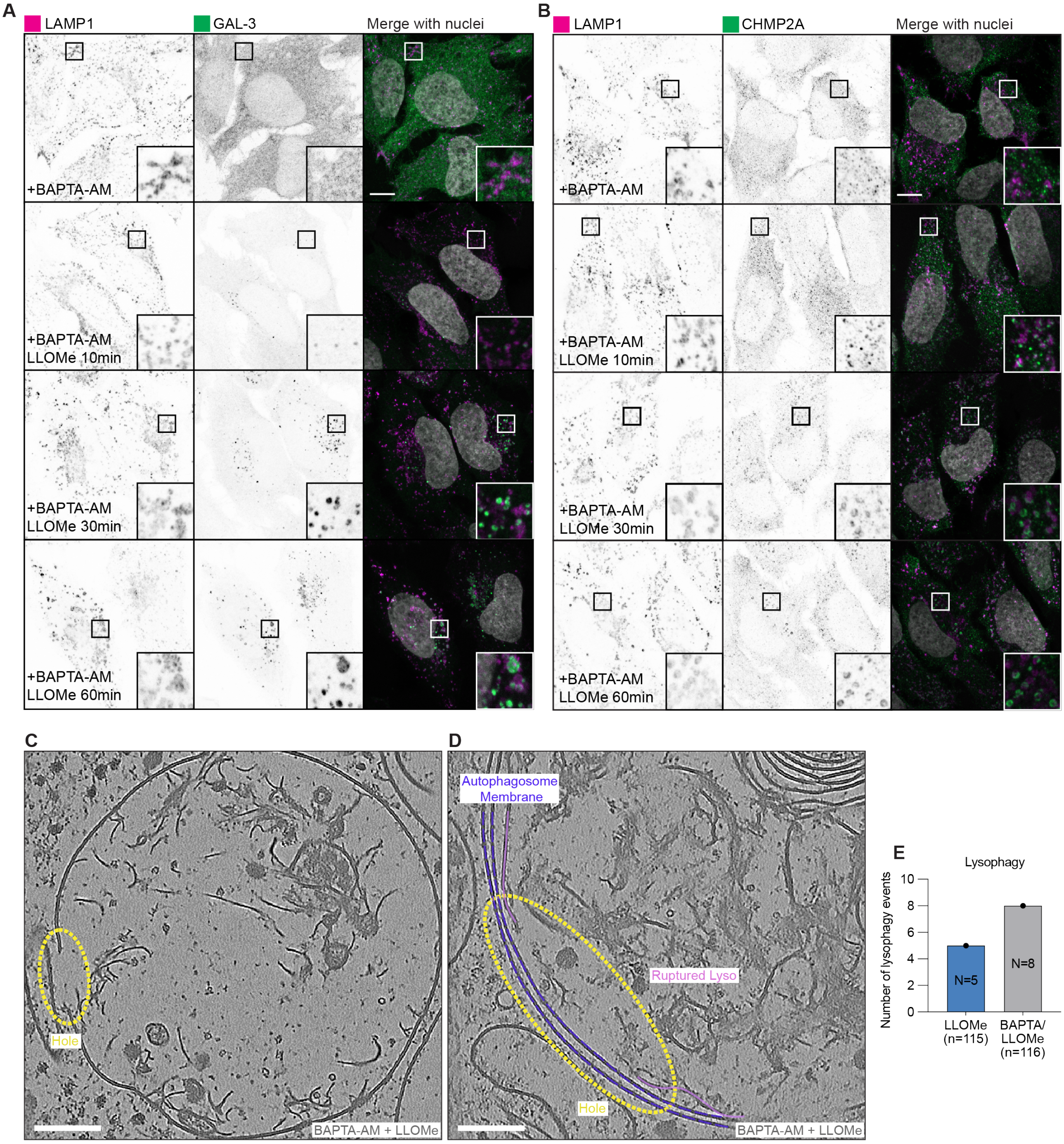

Fig. S3. Ca2+-chelator BAPTA-AM pre-treatment leads to extensive damage.

(**A**) Confocal images of immunofluorescence against LAMP1 (lysosome) and GAL-3 (lysosomal damage marker) with BAPTA-AM pre-treatment (50 μM, 30 min). Split channels and a merged image with Hoechst (nucleus) are shown. Untreated control and timepoints (10, 30, 60 min) after LLOMe treatment (0.5 mM) are indicated. (**B**) Same timepoints and treatments as in A but stained for CHMP2A. Scale for IF panels: 10 μm. (**C**) Tomographic slice of BAPTA-AM (50 μM, 30 min) LLOMe treated (0.5 mM, 60 min) lysosome with an apparent hole (circled in yellow). Scale: 100 nm. (**D**) Tomographic slice of lysosomes in BAPTA-AM+LLOMe treated cells engulfed in an autophagosome (autophagosome membrane outlined in blue, lysosomal membrane in purple). Scale: 100 nm. (**E**) Quantification of lysophagy events observed across LLOMe (N=5, n=115 tomograms) and BAPTA-AM+LLOMe datasets (N=8, n=116 tomograms).

bioRxiv preprint doi: https://doi.org/10.64898/2026.01.17.700056; this version posted January 19, 2026. The copyright holder for this preprint (which was not certified by peer review) is the author/funder, who has granted bioRxiv a license to display the preprint in perpetuity. It is made available under aCC-BY 4.0 International license.

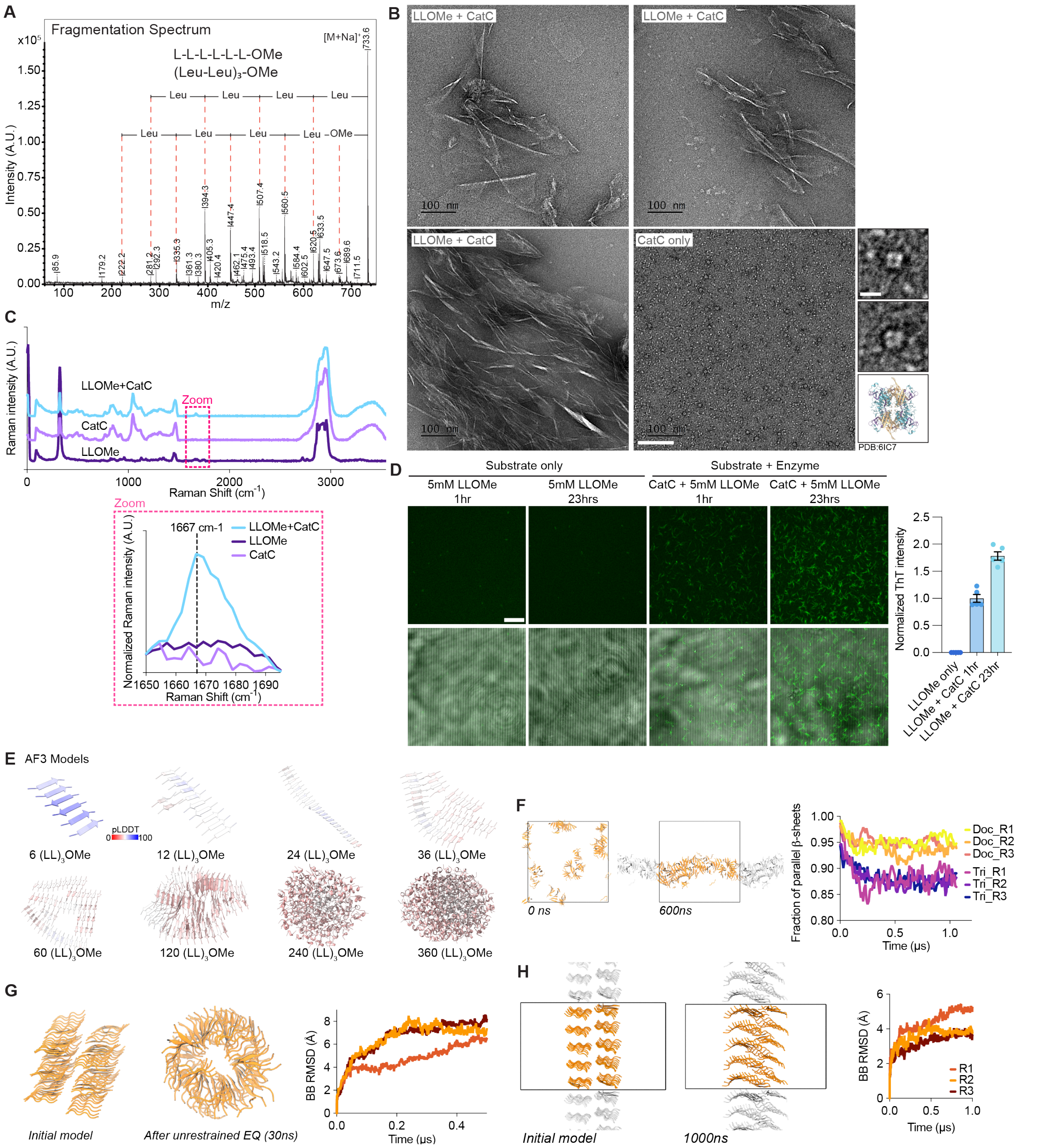

Fig. S4. In vitro reconstitution of LLOMe formed amyloids

(**A**) Fragmentation spectrum of $(LL)_3$OMe peak shown in Fig. 3A indicating the chemical composition of found species. (**B**) Negative Stain EM micrographs of LLOMe (5 mM) with CatC (5 ng/μL) and CatC alone at pH 7.5. Scale: 100 nm and 10 nm for insets. CatC atomic model (PDB:6IC7) shown for comparison. (**C**) Raman spectra of LLOMe (1 mM), CatC (1 ng/μL) and LLOMe+CatC. Background-subtracted, z-score Raman intensities are shown. (**D**) ThT staining of LLOMe amyloids without CatC present (5 mM, 1 h and 23 h) and active CatC (1 ng/μL). Quantification of ThT intensity of LLOMe only and LLOMe+CatC for 1 h and 23 h (n=5, mean ± s.e.m.). (**E**) Alphafold3 models of 6, 12, 24, 36, 60, 120, 240, 360 $(LL)_3$OMe molecules, colored according to AF3 prediction confidence (pLDDT). (**F**) MD simulation of β-sheets fragments spontaneously forming fibril-like structures after 600 ns. Plot showing fraction of parallel β-sheets for pre-formed parallel trimers (Tri) or dodecamers (Doc) at 400 K in 1 μs simulation time over three repeats. (**G**) MD simulation of assembled parallel β-sheets that spontaneously form a fibril and RMSD plot over 0.5 μs to visualize ensemble stability over three repeats. (**H**) Stack of $(LL)_3$OMe assemblies in sheet-like conformation initially and after 1 μs simulation time. BB RMSD plot showing fibrils remaining stable over 1 μs simulation time.

bioRxiv preprint doi: https://doi.org/10.64898/2026.01.17.700056; this version posted January 19, 2026. The copyright holder for this preprint (which was not certified by peer review) is the author/funder, who has granted bioRxiv a license to display the preprint in perpetuity. It is made available under aCC-BY 4.0 International license.

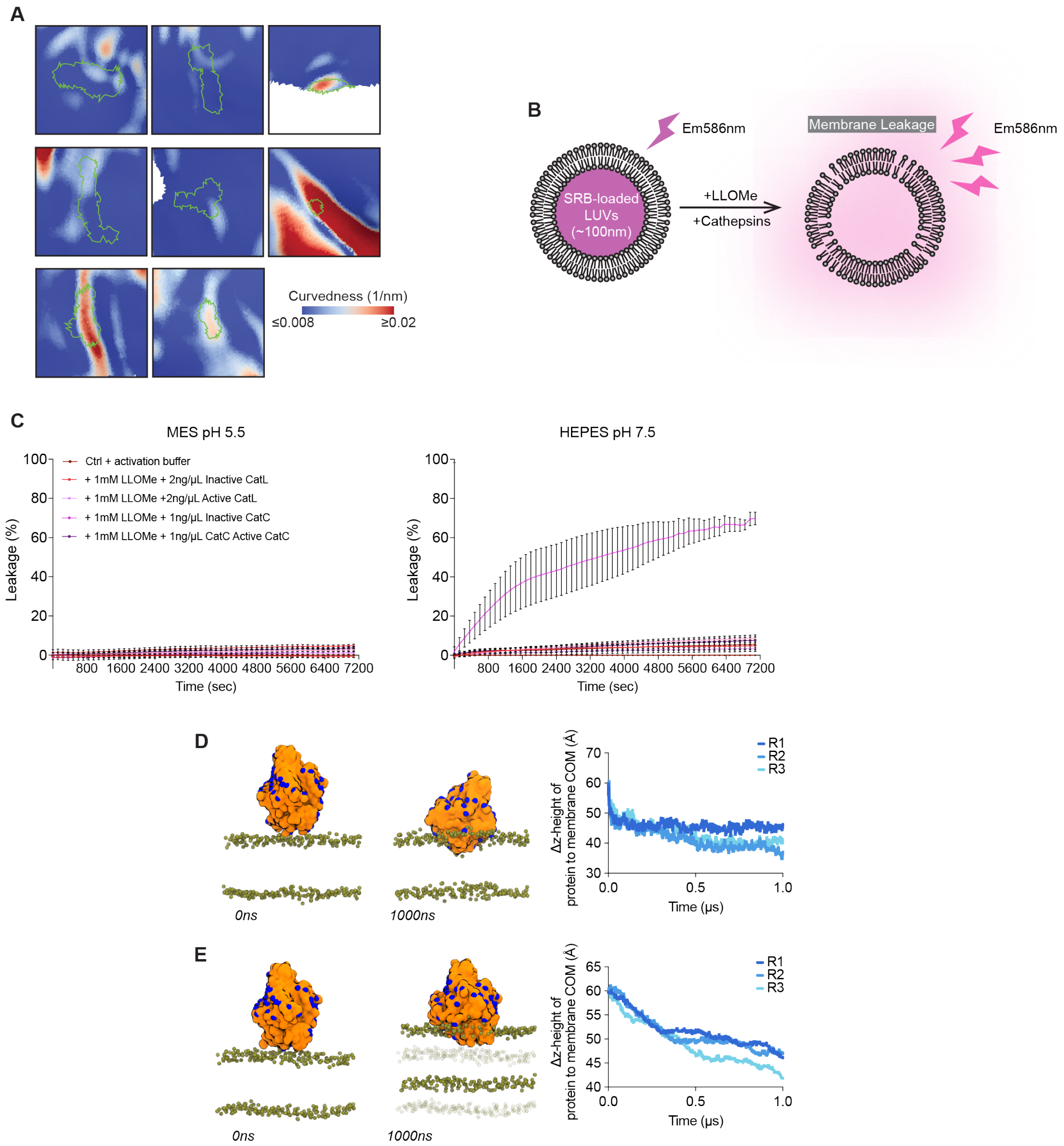

Fig. S5. In vitro and in silico LLOMe fibrils are able to damage membranes

(**A**) Examples of the fibril associated patches (green outline) overlayed with curvedness measurements represented on lysosomal surface mesh. Scale is indicated in figure. (**B**) Schematic of liposome leakage assay mimicking lysosomal damage. SRB-fluorescence was used as readout of membrane leakage. (**C**) Percentage of leakage of liposomes at pH 5.5 (left) and pH 7.5 (right) across all conditions and time (n=3, mean ± s.e.m.). (**D**) MD simulation of LLOMe fibril (orange) freely moving in simulation box with membrane bilayer. Phosphate atoms are colored green, fibril's nitrogen at N-termini is colored in blue. Initial fibrils position is shown vs. fibril position after 1000 ns. Plot showing z-distance between protein and membrane center of mass (COM) over three repeats. (**E**) MD simulation of LLOMe fibril (orange) fixed on distal end towards membrane. Initial position is shown vs. position after 1000 ns. Initial phosphate atom positions are shown in transparent. Plot showing z-distance between protein and membrane COM for three repeats.

bioRxiv preprint doi: https://doi.org/10.64898/2026.01.17.700056; this version posted January 19, 2026. The copyright holder for this preprint (which was not certified by peer review) is the author/funder, who has granted bioRxiv a license to display the preprint in perpetuity. It is made available under aCC-BY 4.0 International license.

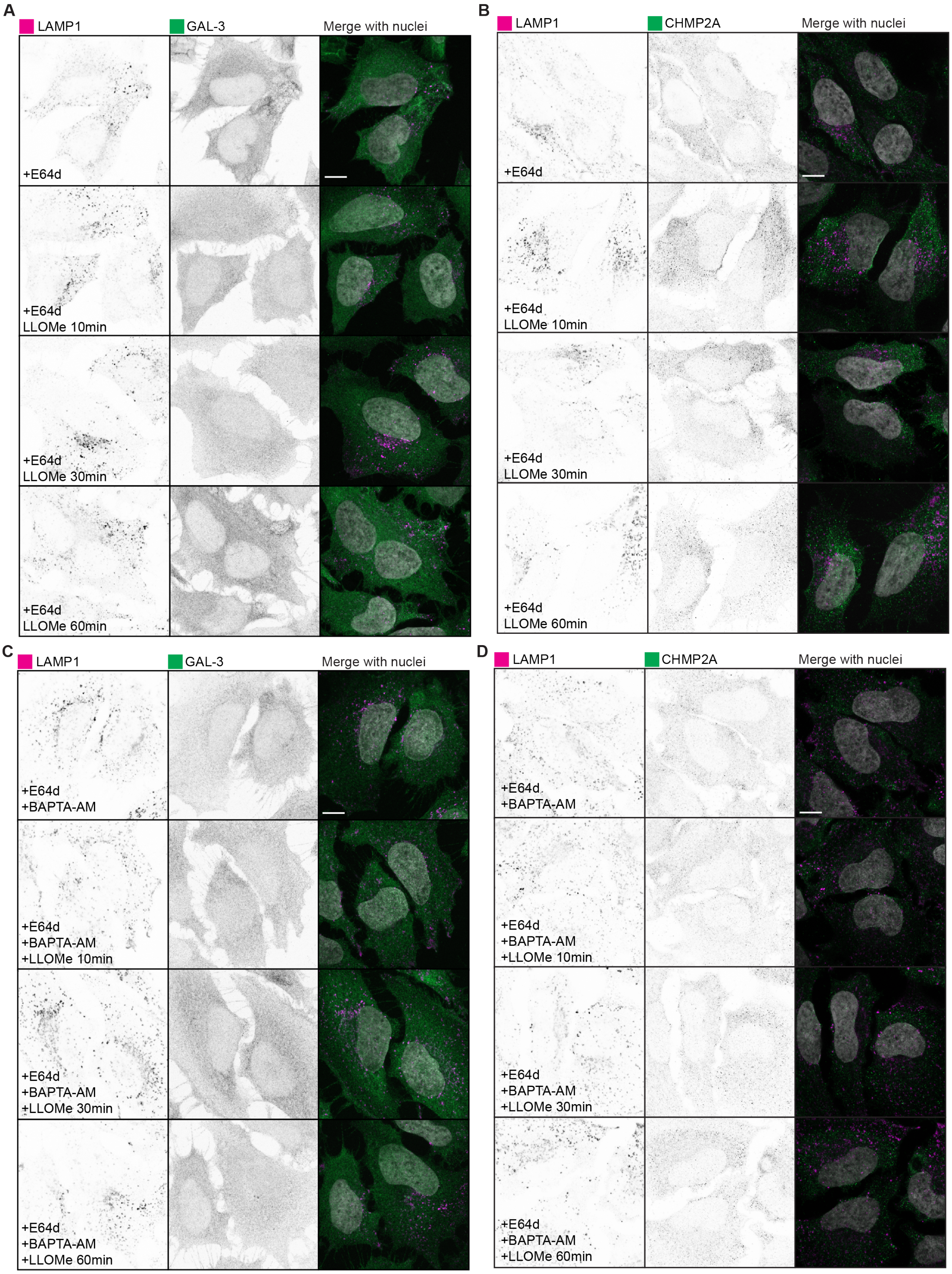

Fig. S6. Cellular responses to E64d ±BAPTA-AM before LLOMe-treatment.

(**A**) Confocal images of immunofluorescence against LAMP1 (lysosome) and GAL-3 (lysosomal damage marker) with E64d pre-treatment (100 μM, 30 min). Split channels and a merged image with Hoechst (nucleus) are shown. Untreated control and timepoints (10, 30, 60 min) after LLOMe treatment (0.5 mM) are indicated. (**B**) Same timepoints and treatments as in A but stained for CHMP2A. (**C**) Confocal images of immunofluorescence against LAMP1 (lysosome) and GAL-3 (lysosomal damage marker) with E64d (100 μM, 30 min) +BAPTA-AM (50 μM, 30 min) pre-treatment. Split channels and a merged image with Hoechst (nucleus) are shown. Timepoints (10, 30, 60 min) after LLOMe treatment (0.5 mM) are indicated. (**D**) Same timepoints and treatments as in C but stained for CHMP2A. Scale for IF panels: 10 μm.

bioRxiv preprint doi: https://doi.org/10.64898/2026.01.17.700056; this version posted January 19, 2026. The copyright holder for this preprint (which was not certified by peer review) is the author/funder, who has granted bioRxiv a license to display the preprint in perpetuity. It is made available under aCC-BY 4.0 International license.

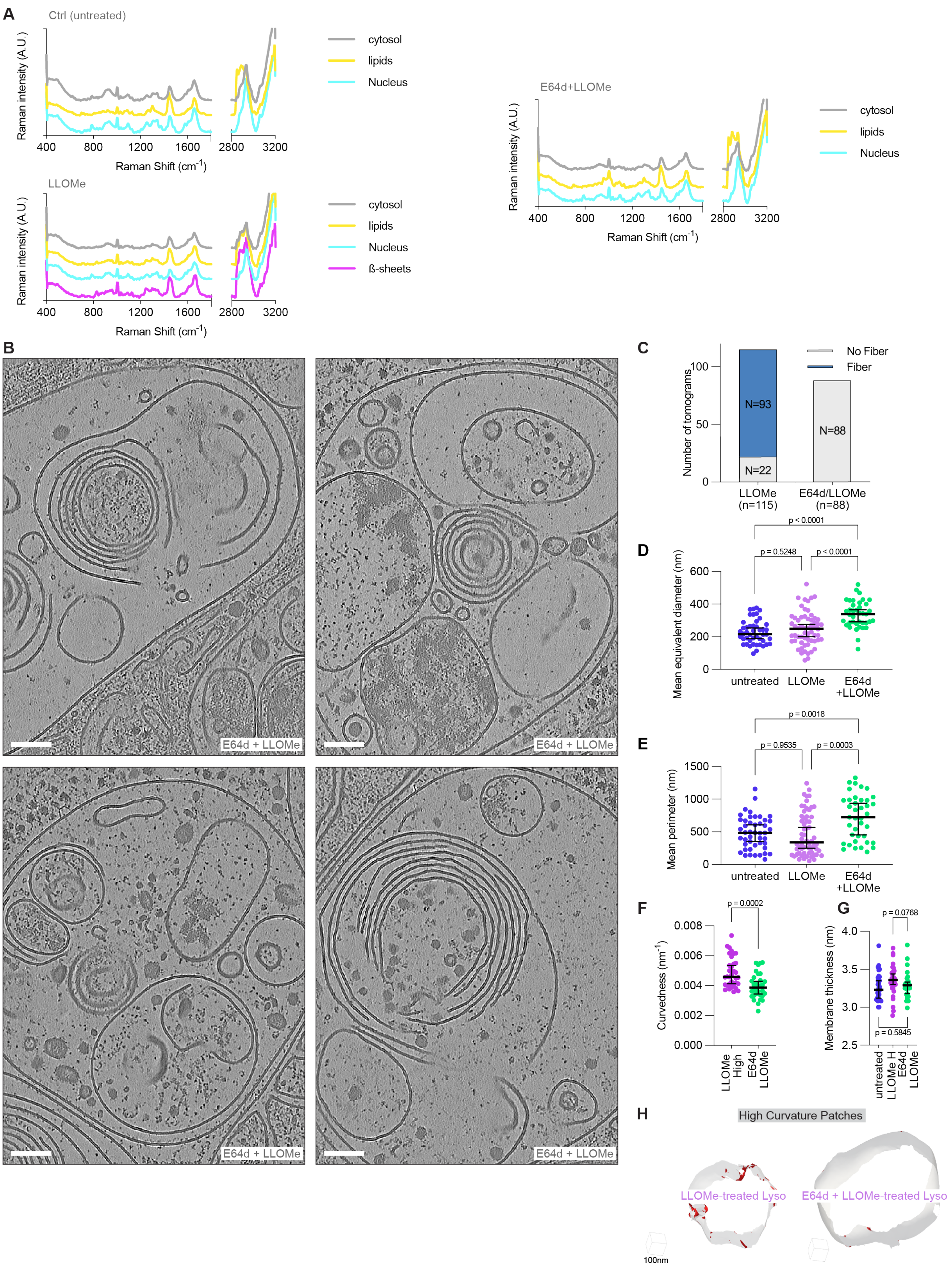

Fig. S7. E64d negates LLOMe-amyloids by inhibiting CatC

(**A**) Raman spectra of cells shown in Fig. 4M (untreated, LLOMe, and E64+LLOMe). Spectra of nucleus, cytosol, lipids, and β-sheets-regions are visualized separately. Between 1800 cm^{-1} and 2800 cm^{-1} no peaks could be detected therefore x-axis was cut for visual purposes. (**B**) Tomographic slice of lysosomes in E64d (50 μM, 30 min) LLOMe (0.5 mM, 60 min) treated cells. Scale: 100 nm. (**C**) Quantification of tomograms with amyloid fibrils (N) inside lysosomes between with (n=88) and without (n=115) E64d pre-treatment before LLOMe. (**D**) Mean equivalent distance measurements of lysosomes from three different datasets (untreated (n=46), LLOMe (n=60), E64d+LLOMe (n=39)). (**E**) Mean perimeter measurements. Individual data points are indicated, median with 95% confidence interval drawn. Statistical test: one-way ANOVA with multiple comparisons test, p-values are indicated. (**F**) Morphometric analysis of E64d+LLOMe tomograms for general organelle curvedness. Comparison between high-damaged LLOMe lysosomes and E64d+LLOMe lysosomes. (**G**) Comparison of membrane thickness between untreated, highly-damaged LLOMe, and E64d+LLOMe lysosomes. (**H**) Lysosome membrane segmentation rendered with red highlights for areas curved above 0.021 nm^{-1}.

bioRxiv preprint doi: https://doi.org/10.64898/2026.01.17.700056; this version posted January 19, 2026. The copyright holder for this preprint (which was not certified by peer review) is the author/funder, who has granted bioRxiv a license to display the preprint in perpetuity. It is made available under aCC-BY 4.0 International license.

Movie S1.
In situ cryo-ET tomogram of LLOMe-damaged lysosome shown in Fig. 1F (0.5 mM, 60 min).

Movie S2.
MD simulation rendering of fixed fibril inserting into lysosome-like membrane, shown in Fig. S5E. Simulation time: 1 μs. Fibril is colored in orange, phosphates in green. The original positions of the membrane phosphates are shown as transparent spheres.

Movie S3.
MD simulation rendering of fibril inserted in membrane during equilibration phase, creating holes in both sides of the lysosome-like membrane, shown in Fig. 4I. Simulation time: 1 μs. Fibril is colored in orange, phosphates in green, and water molecules are colored in licorice. Na^+ (yellow) and Cl^- (lightblue) ions are displayed as spheres.
